# How do the smart travel ban policy and intercity travel pattern affect COVID-19 trends? Lessons learned from Iran

**DOI:** 10.1371/journal.pone.0276276

**Published:** 2022-10-18

**Authors:** Habibollah Nassiri, Seyed Iman Mohammadpour, Mohammad Dahaghin

**Affiliations:** Civil Engineering Department, Sharif University of Technology, Tehran, Iran; Tsinghua University, CHINA

## Abstract

COVID-19, as the most significant epidemic of the century, infected 467 million people and took the lives of more than 6 million individuals as of March 19, 2022. Due to the rapid transmission of the disease and the lack of definitive treatment, countries have employed nonpharmaceutical interventions. This study aimed to investigate the effectiveness of the smart travel ban policy, which has been implemented for non-commercial vehicles in the intercity highways of Iran since November 21, 2020. The other goal was to suggest efficient COVID-19 forecasting tools and to examine the association of intercity travel patterns and COVID-19 trends in Iran. To this end, weekly confirmed cases and deaths due to COVID-19 and the intercity traffic flow reported by loop detectors were aggregated at the country’s level. The Box-Jenkins methodology was employed to evaluate the policy’s effectiveness, using the interrupted time series analysis. The results indicated that the autoregressive integrated moving average with explanatory variable (ARIMAX) model outperformed the univariate ARIMA model in predicting the disease trends based on the MAPE criterion. The weekly intercity traffic and its lagged variables were entered as covariates in both models of the disease cases and deaths. The results indicated that the weekly intercity traffic increases the new weekly COVID-19 cases and deaths with a time lag of two and five weeks, respectively. Besides, the interrupted time series analysis indicated that the smart travel ban policy had decreased intercity travel by around 29%. Nonetheless, it had no significant direct effect on COVID-19 trends. This study suggests that the travel ban policy would not be efficient lonely unless it is coupled with active measures and adherence to health protocols by the people.

## Introduction

COVID-19 (COVID) was first detected in Wuhan, China, on December 31, 2019; the local epidemic transformed into a worldwide pandemic rapidly. Iran was the first Middle East country which reported a COVID death. Since then, the country has been largely affected, with more than seven million confirmed cases and over one hundred thousand COVID deaths [1]. In response, the governments imposed social distancing and lockdown policies to contain the pandemic. Which in turn resulted in new challenges such as the economic recession [2], mental health issues [3], educational concerns [4], and food insecurity concerns [5]. In contrast to the dark side of the epidemic, the reduction of road traffic and polluting activities has led to positive environmental and ecological results [6]. Besides, some studies reported significant declines in road traffic fatalities [7].

Nowadays, infectious diseases are spreading around the world significantly at a sharper pace. This fact could be referred to the ease of international travel and increased populated metropolitans [8]. COVID is a viral illness with no definitive treatment, and therefore, controlling the transmission of the disease is the main way to contain the pandemic. China’s government has been successful in controlling the disease in its first stages by enforcing strict quarantine policies on infected individuals and cities, as well as controlling intercity traffic [9]. On the other hand, studies illustrated that the travel quarantine of Wuhan delayed the overall epidemic progression by only 3 to 5 days in Mainland China [10]. Moreover, the modelling study showed that additional travel limitations, up to 90% travel restrictions to and from Mainland China, only modestly affect the epidemic trajectory worldwide unless combined with a 50% or higher reduction of transmission in the community [10]. Consequently, it is crucial to inform people that a reduction in mobility does not necessarily lead to a decline in COVID trends and that it is therefore essential for citizens to take active measures to protect themselves and others. In addition to the often-inconsistent results of previous studies, some developing countries do not have the economic strength to adopt strict quarantine and travel ban policies. So, such countries can employ solely certain elements of China’s strategy, including suspension of public transportation, closure of recreational areas, and a ban on public assemblies that have been the most effective measures to contain the disease [11].

Iran employed a very flexible policy in both international and intercity travels. On March 27th, 2020, authorities implemented a 1.8-meters social distancing policy on COVID in all Iranian cities to control the disease. To preserve the economy, the public health authorities divided the business activities into four groups, and a smart, dynamic policy was adopted so that specific groups would not be allowed to operate in the corresponding specified severity levels of the epidemic. An interrupted time series analysis indicated the efficiency of the policy as it has declined the COVID’s deaths during the first peak significantly [12]. On November 21, 2020, the government applied a new intervention, termed the “Smart Travel Ban (STB)” policy. According to this policy, only drivers who intend to travel intercity for commercial and essential purposes can commute. Private car drivers will face significant fines if they violate the travel ban on the intercity highways. Besides, the travel ban is smart and dynamic as it is implemented between cities with certain epidemic severity levels, and is not constant over time and space. To date, no study has examined the effectiveness of the policy in reducing intercity travel and COVID trends in Iran.

The multivariate time series regression models could be of great help in making well-informed decisions for the health policymakers in controlling the spread of infections and managing the over-stressed health infrastructures [13]. The COVID affects the respiratory system in the patients, which higher the demand for intensive care units (ICU). Consequently, it is crucial to predict peaks of demand accurately to provide adequate infrastructure. Moreover, dynamic forecasting models are also employed to analyze and compare the impacts of various implemented interventions. Besides, another advantage of multivariate time series analysis is their ability to map real solutions by illustrating the underlying time-varying influential factors. In addition to the number of adopted variables, epidemic modelling methods have evolved and can be widely classified into three categories, mathematical, statistical, and advanced analytical modelling techniques [14]. The traditional mathematical techniques such as the SEIR (Spread, Exposed, Infected, and Recovered) model are the most employed methods in epidemiology. These techniques are inherently deterministic and cannot correctly handle the spatiotemporal variability and uncertain nature of the COVID pandemic in the long-term predictions [15]. Advanced analytical models such as machine learning, deep learning, and artificial neural networks have been widely employed to account for the nonlinear dependencies in the time series. The theoretical basis of these models in time series prediction has almost been questionable. Because it is not clear that if the trend and seasonal components are detected, should they be discarded before model estimation or not? Moreover, the best approach to split the dataset to training and test data is not clear in the literature [16]. The most utilized statistical time series regression model is the ARIMA method which has indicated its advantages in capturing the linear dependence in the time series and is one of the best linear models for such data [17]. Indeed, the advanced nonlinear models only outperform the ARIMA method when the nonlinear dependence is evident in the time series data set [18].

This study aimed to suggest answers to the following questions:

1. How has the smart travel ban policy influenced the intercity travel pattern and COVID trends (e.g., new weekly COVID cases and new weekly COVID deaths) in Iran?
2. Does the multivariate ARIMAX model outperform its univariate counterpart in COVID trend prediction, incorporating the simultaneous effects of exogenous variables?

This research adds to the body of knowledge on transport policy-making and containing the COVID pandemic. First, the efficiency of the smart travel ban policy has not been examined to date. The economy-friendly and dynamic features of this policy make it practical for implementation in Low-to-Middle-Income Countries (LMICs). Second, the multivariate COVID forecasting tools can more accurately capture the underlying trends. That is while all the past studies have used the univariate analysis framework in Iran. Third, there is still limited knowledge on the quantitative effects of intercity travel patterns on COVID trends, as well as on the duration of time that should be used to capture changes after policy implementation on controlling human mobility.

The rest of the paper is organized as follows. A thorough literature review of previous studies is presented in section 2. The details of the related dataset and the methodology of developed models are provided in the next section. The models are fitted to the data, and the forecasting results are presented in Section 4 and discussed in Section 5. Finally, the paper is concluded in Section 6.

## Literature review

### The association of human mobility & COVID-19 trends

Xiao et al. [9] reported that the quarantine policy declined the effective reproductive numbers (***R***_***t***_) from a maximum of 3.98 to below 1 in the first wave of the pandemic in China. According to the results of another study, the cumulative cases in the 371 cities of China showed a significant relationship with the population inflows from Wuhan. For each 1% increase of population inflows, the number of confirmed cases is predicted to increase by 5.98%. Consequently, the quarantine policy has been effectively reduced the rate of disease spread within the country [19]. Besides, studies indicated that the continuous UK government’s guidance to avoid non-essential travel, combined with the closure of schools and reduced operation of London Underground and national railway services, contributed to a continuous reduction in human mobility and effectively contained the pandemic during the first wave [20]. Meng et al. [21] developed a hybrid seasonal ARIMA intervention model to analyze the impacts of various control measures employed in the USA, China, and Singapore on air travel during the pandemic. They reported that the short-term adverse economic effects of stricter and more effective measures are large, but the long-term impacts would be milder. Another study investigated the effectiveness of anti-COVID measures in Poland and their impacts on public transport. Results indicated that the interventions had strongly decreased the public transport mode choice. Besides, the government restrictions and media campaigns were more influential on human mobility than the information on daily new COVID cases [22]. Cowling et al. [23] explored the impacts of Hong Kong policies, including the travel ban, school closure, and public behavioural changes. They stated that the daily effective reproduction number remained around one during the policies implementation period, which denotes the measure’s effectiveness. On the other hand, past experiences during the 2003 SARS outbreak in Singapore also indicated that strict travel limitations have a slight effect unless paired with public health interventions and behavioural changes that achieve a considerable reduction in the disease transmissibility [24]. A comparative study was conducted on implemented COVID interventions in the six developed countries, containing 418 policies, of which 244 were transport measures. The results illustrated that none of the COVID interventions in public health and transport is associated with a decline of cumulative deaths or cumulative infection cases. Solely 40% of measures were detected meaningful in reducing the daily new cases [25].

### Methodological approaches

Since the emergence of the pandemic, studies have utilized various methods to predict the COVID trends and identify the underlying factors. Choi and Ahn [26] predicted the daily imported COVID cases in South Korea, using the daily mobile roaming data as the covariate. The ARIMAX modelling technique outperformed the univariate ARIMA model based on the forecast accuracy measures. The roaming data was associated with imported COVID cases with a time lag of 12 days. Shao et al. [27] conducted mediation effect analysis using linear regressions. The study illustrated that temperature affects the transmission rate of COVID, influencing human mobility. The temperature influenced the transmission rate by a time lag of 1 to 14 days. Ray et al. [28] implemented an extension of the SIR model with a Bayesian hierarchical structure, where SIR stands for Susceptible, Infected and Recovered and illustrates the three feasible states of the members of a population afflicted by a contagious disease. The study suggested that at least 42 days of lockdown would be needed to reduce the cumulative COVID cases in India. A comprehensive comparative study employed three growth curve-fitting models, two mathematical models (SEIR & IDEA), two statistical models (ARIMA, Holt’s exponential), and four machine/deep learning models (Neural Network, LSTM Networks, GANs, and Random Forest) on ten COVID datasets of prominent regions worldwide [14]. The study indicated that the machine/deep learning models almost reveal poor performance since the small size and low complexity of the data reduces the chances of effectively training the models. On the other hand, Holt’s exponential model and ARIMA almost outperformed other models. Nonetheless, Holt’s procedure only worked well when low trends were seen in the data without other components like cyclicity, seasonality, and randomness.

Several studies have also analyzed the COVID trends in Iran. Tran et al. [29] analyzed the daily time trends of infections and death in Iran using the ARIMA model. The study predicted the disappearance of the first wave of the epidemic. Other studies also employed the univariate ARIMA method, analyzing the daily COVID trends in Iran [30, 31]. They predicted an exponential increase for COVID trends during the first wave. Moftakhar et al. [30] compared the forecast accuracy of ARIMA with artificial neural networks (ANNs) in predicting the total daily infections in Iran. The results indicated that ARIMA significantly outperforms the ANNs. Shen [32] adopted a logistic growth model on daily COVID cases in Iran, South Korea, and China. Zarie et al. [11] developed a SIR epidemiological model to estimate the COVID infections for the upcoming month. The authors adopted the transmission rate, recovery rate, and mortality rate parameters from China’s outbreak. Besides, the classical SEIR model has also been applied in Iran [33]. Talkhi et al. [34] compared the performance of ARIMA, Holt-Winters, Prophet, multilayer perceptron, and extreme learning models. Holt’s exponential model revealed the best fit for Iran. Another study predicted the COVID new cases using machine-learning methods. They stipulated that the pandemic’s peak occurs 150 days after the outburst [35]. Kafieh et al. [36] suggested the LSTM deep learning method, predicting COVID trends in Iran. Studies also utilized the Gompertz and von Bertalanffy mathematical growth models to predict the number of hospitalizations in Iran [37]. Despite the importance of multivariate time series analysis in the precise prediction of COVID trends and identifying the simultaneous impacts of influential underlying factors, no study has adopted the dynamic multivariate forecasting framework in Iran. Besides, there are limited studies that analyzed the association of human mobility and COVID trends worldwide [27, 38–40].

## Materials and methods

### Data processing

This research utilized the epidemiological data from the Johns Hopkins University Center for Systems Science and Engineering COVID dashboard, which includes daily data on COVID cases and deaths for all countries [1]. The Ministry of Roads and Urban Development (MRUD) reports the traffic flow in intercity highways of Iran, recorded by loop detectors. The daily traffic counts on rural highways of Iran were also collected from the dataset of MRUD. The intercity travel pattern follows the holidays based on the Persian Calendar. So, the whole data were aggregated weekly, based on the Iranian calendar (the Solar Hijri calendar). Finally, the time series of weekly new cases and mortalities due to COVID disease and the weekly traffic flow from Esfand 3, 1398 (February 22, 2020) to Shahrivar 12, 1400 (September 3, 2021) were analyzed (e.g., 80 data points). The first 70 data points were used as the training sample, while the remaining data were adopted as the test dataset.

The models were estimated in Eviews version 10, using the maximum likelihood method. The Augmented Dickey-Fuller (ADF) test was also calculated by Eviews software. Besides, the Ljung-Box (LB) and Kolmogorov-Smirnov (KS) tests on the residuals, ACF/PACF, and time series plot analysis was conducted by Minitab version 19.

### Methodology

Box and Jenkins [41] developed the ARIMA model, which combines the autoregressive (AR) and moving average (MA) models. Also, the differencing is explicitly included in the formulation. The AR model describes a time series in which the current observation depends on its preceding values, whereas the MA model describes a time series as a linear function of current and previous random errors [42]. The structure of the model is as **ARIMA** (**p, d, q**), where ***p*** is the autoregressive order, ***d*** is the number of nonseasonal differencing operations, and the ***q*** is moving average order. The ARIMA model in terms of lag polynomials is written as follows [43]:

$$\varphi_{p}(L)(1-B{)}^{d}y_{t}=\theta_{q}(L)\varepsilon_{t}$$ (1)

where (**1**−***B***)^***d***^***y***_***t***_ denotes the d order regular differenced dependent variable, removing non-stationarity from the series, and ***ε***_***t***_ is the random error at time period ***t***. The ***φ***_***p***_(***L***), and ***θ***_***q***_(***L***) terms are the AR(P) and MA(q) models, respectively.

ARIMAX model [44] extends the ARIMA model, adding explanatory value to its counterpart. Scholars refer to the ARIMAX model as “dynamic regression” since it accounts for the dynamic impacts of exogenous variables [45]. The model is defined as follows [46]:

$$y_{t}=\varpi_{0}I_{t}+\beta X+N_{t}$$ (2)

Where;

$$N_{t}=\frac{\theta_{q}(B)u_{t}}{\varphi_{p}(B)(1-B{)}^{d}}$$ (3)

In which ***y***_***t***_ is the proper transformation of the dependent variable; ***I***_***t***_ is the intervention component, X is an array of exogenous variables, and ***N***_***t***_ represents the error term, denoted by ***ARIMA*** (***p*, *d*, *q***) model. Besides the ***φ***_***p***_, and ***θ***_***q***_ components are the AR(p) and MA(q) operators, and ***B*** is the backshift operator. The ***u***_***t***_ is a Gaussian white noise error term.

The Box-Jenkins algorithm contains a three-step iterative modelling procedure, namely model identification, parameter estimation, and diagnosis checking [41]. The stationarity is one of the primary assumptions of ARIMA models, meaning that the statistical properties of the series, such as mean, variance, and autocorrelation, should all be constant over time [42].

Firstly, the central assumption of stationarity of the time-series should be checked based Augmented Dickey-Fuller (ADF) [47] test. The null hypothesis of the ADF test is that there is a unit root for the series, which in turn confirms the presence of non-stationarity in the series. The non-stationarity problem could almost be handled simply by applying differencing. Moreover, the visual inspection of the time series and ACF/PACF plots are also employed to check for stationarity. The next step is the model identification, when the order of parameters of the SARIMA model is identified, investigating the autocorrelation function (ACF) and the partial autocorrelation function (PACF) plots. After model postulation, the parameters are estimated based on the maximum likelihood approach. Then, the candidate models are ranked based on the lowest BIC and MAPE criteria.

The final step is diagnostic checking. If the model accurately explains the series, the residuals will represent white noise characteristics, containing zero-mean, constant variance, and independence. The white noise properties of the residuals are visually inspected using the ACF/PACF residual plots. Also, the independence and normality properties of residuals should be tested based on the Ljung and Box (LB) and Kolmogorov-Smirnov (KS) tests, respectively. The LB test [48] is general goodness of fit statistic. Indeed, it checks for the goodness of fit of the ACF function of residuals to the ACF of the white noise process. Hence, if the LB statistic is not statistically significant, the null hypothesis of the white noise properties of residuals will not be rejected. Finally, the observed and predicted weekly COVID cases and deaths are compared to test the models’ forecast accuracy using the test dataset.

## Results

To investigate the effectiveness of the smart travel ban (STB) policy, the interrupted time series analysis framework was adopted. Firstly, the Box-Jenkins methodology was applied to develop a traffic flow prediction model. Then the policy implementation date (the 40th week) was entered as the intervention variable in an ARIMAX modelling framework to evaluate the significance of the policy in the reduction of weekly intercity travels. Secondly, multivariate COVID dynamic regressions were estimated. The intercity traffic flow and various lagged variables of weekly new confirmed cases were considered in the COVID mortality prediction model as exogenous variables. On the other hand, the intercity traffic was the only regressor in the new COVID cases model. Finally, the COVID forecasting tools were utilized to investigate the significance of the direct effects of the STB policy on the underlying COVID trends.

Our main objective is to identify the best forecasting models for the weekly new cases and deaths time-series datasets. To compare and validate the models, each dataset was separated into two parts. The first 70 data points from Esfand 3, 1398 (February 22, 2020) to Tir 4, 1400 (June 25, 2021) were used as the training sample to fit the models. The subsequent ten weeks were employed as the testing sample for assessing the models’ forecast accuracy. Finally, the performance of the models was compared, using the lower Bayesian information criterion (BIC), the lower mean absolute percentage error (MAPE), and the higher coefficient of determination as accuracy measures.

### Smart travel ban policy & intercity travel pattern

**Fig 1:**
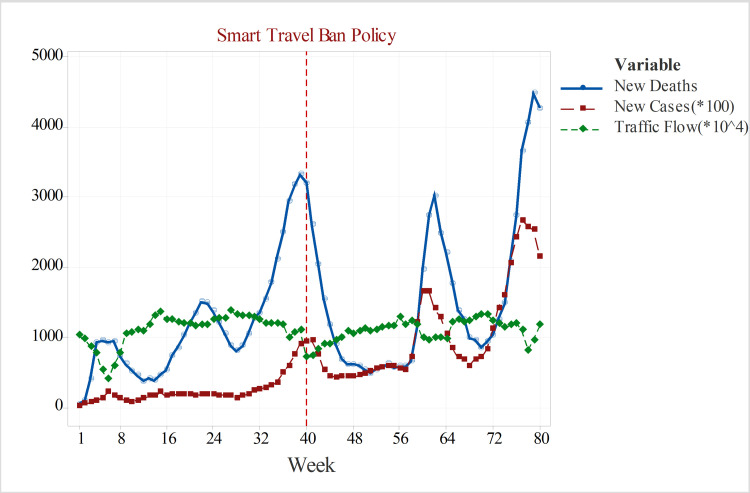
Weekly new COVID deaths and cases (*10^2^) and weekly intercity traffic flow (*10^4^) in Iran.

**Fig 2:**
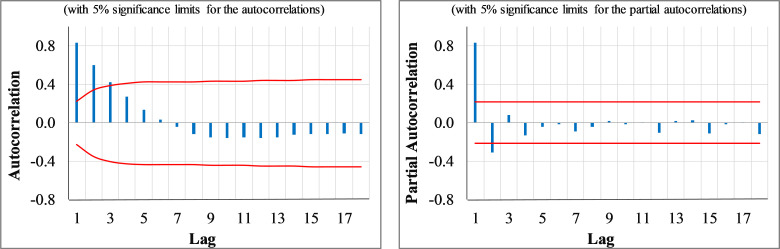
The ACF and PACF plots of the log-transformed weekly intercity traffic time series.

The time series plot of the weekly intercity traffic is illustrated in Fig 1. The figure illustrates that after the policy implementation, a reduction in intercity travels is evident. Although there is no evidence for the non-stationarity in the mean or variance, the stationarity test was implemented. The ADF test did not reject the null hypothesis of non-stationarity in the original series (see Table 1). Hence, a Box-Cox transformation was applied with λ = 0 to stabilize the mean and variance. The resulted series was stationary as the ADF test rejected the test’s null hypothesis at a 90% confidence level (see Table 1). Fig 2 indicates the ACF/PACF plots of the transformed series. The ACF plot denotes an exponential decay form. Besides, the PACF plot tails off after the first two significant spikes, suggesting that the series follows a pure AR (2) process. To ensure the adequacy of the identified model, various competing ARIMA models were developed, but none of the estimated alternatives had statistically significant parameters. Finally, the only fit models with significant parameters were the ARIMA (2,0,0) and the ARIMA (2,0,0) with the intervention dummy variable (ARIMAX) models. The estimated models are detailed in Table 2.

**Table 1:** P-values of statistical tests for stationarity.

Time series	ADF test
Weekly intercity traffic flow	0.13
Log (Weekly intercity traffic flow)	0.10
Weekly new COVID cases	0.05
Weekly new COVID deaths	0.00

**Table 2:** Intercity traffic flow prediction model for log transformed time-series.

			ARIMA		ARIMAX	
			Coef.	t-stat	Coef.	t-stat
*Explanatory variables*						
Intervention variable (*I*_*t*_).			-	-	**-0.149**	**-3.70**
AR (1)			1.138	12.68	1.220	16.15
AR (2)			-0.335	2.67	-0.332	-3.71
Constant			7.030	160.15	7.110	115.65
*Descriptive statistics*						
Length of series			70		80	
Log-likelihood			113.73		133.53	
*Accuracy (within sample)*						
Bayesian information criterion (BIC)			-3.007		-3.064	
Mean absolute % error (MAPE)			0.433		0.433	
Mean absolute deviation (MAD)			0.030		0.030	
Root mean square error (RMSE)			0.047		0.045	
**R** ^**2**^			**0.763**		**0.767**	
*Diagnosis Check of Residuals*						
Ljung and Box (LB) (K = 18)			1.17	0.14	7.61	0.55
Kolmogorov-Smirnov (KS)			0.15	<1.46	0.13	<1.46
*Forecast Accuracy*						
Out of sample MAPE (%)			0.429		-	
*Likelihood Ratio* (*H*_0_: *I*_*t*_ *Coef*. = 0)			39.6	>	*χ*^2^_*α* = .01,*df* = 1_ = 7.87	

Reviewing Table 2, all the independent variables are statistically significant at a 1% significance level. Moreover, the ARIMA model reveals an R-squared value of 0.763, which denotes that the current values of traffic flow could be well explained only by considering the autocorrelations in the time series. Besides, the likelihood ratio test illustrates that the ARIMA model with the intervention variable (ARIMAX) outperforms the univariate model, which confirms the efficiency of the STB policy in reducing intercity travel. Moreover, the MAPE criterion demonstrates that the developed instruments properly reconstruct observations with less than 1% mean absolute error in train and test samples. The t-test of the intervention variable confirms a significant reduction in the weekly intercity traffic after the policy implementation. The coefficient value of the intervention dummy is found to be -0.149, indicating that the introduction of the STB policy reduces the intercity traffic by about 29% if all other factors remain constant.

**Fig 3:**
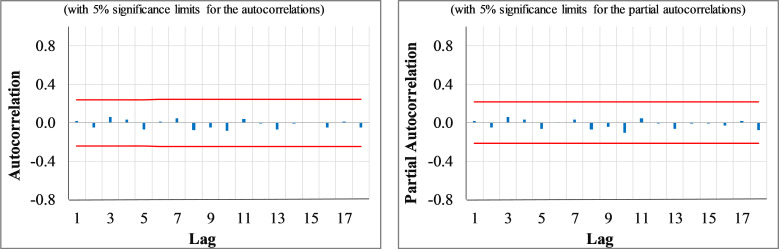
The ACF and PACF plots of the residuals of the ARIMA (2,0,0) intercity traffic prediction model.

Regarding the model diagnosis check, the Gaussian white noise distribution assumption of the residuals was investigated. The ACF/PACF plots of the residuals validate the adequacy of the ARIMA (2,0,0) model (see Fig 3). The figure indicates no significant ACF/PACF coefficients at any lag, meaning no serial correlation exists among the residuals. Besides, the KS test does not reject the null hypothesis of the normal distribution of the error term (see Table 2). Consequently, the developed models capture the underlying process properly. The LB test also confirms the overall adequacy of the developed models, as the test statistics are not statistically significant at a 5 percent significance level (see Table 2).

### Dynamic regressions for weekly COVID new deaths time-series

Fig 1 illustrates the weekly trends of new COVID cases/deaths and intercity traffic flow in Iran. The off-peaks of the intercity traffic are almost associated with peaks of COVID infections and deaths. This fact is related to the dynamic properties of the travel ban policy in Iran. Indeed, whenever the COVID pandemic peaks up, the travel bans automatically become stricter, which in turn results in a gradual decay of the pandemic wave with a time lag. One of the main objectives of this study is to find out this time lag that is crucial information for health authorities to evaluate the effectiveness of the intercity travel ban policies in containing the pandemic. Fig 1 also illustrates that the weekly COVID deaths time series follows the new COVID cases with a time lag of one to two weeks. Hence, considering the lagged variables of weekly COVID cases in the COVID mortalities prediction model would be wise advice.

**Fig 4:**
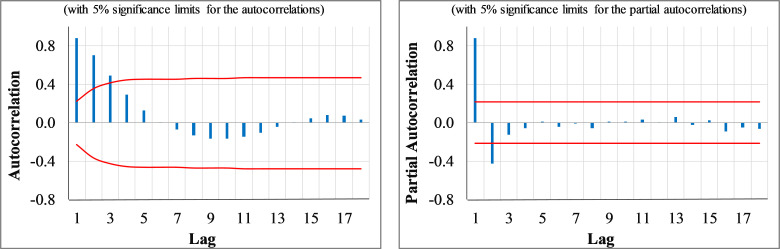
The ACF and PACF plots of the original weekly new COVID deaths time series.

The time series plot of the weekly COVID mortalities suggests that the time series would be stationary in mean and variance. Since no significant trend or fluctuations in the variance is detected over time. Also, the ADF test rejects the null hypothesis of non-stationarity at a 1% significance level for the COVID deaths time series (see Table 1). Fig 4 indicates the ACF/PACF plots of the original series for the first 18 lags (e.g., a quarter of the total period [49]. The ACF plot exhibits an exponential decay behaviour, and only the two first lags’ autocorrelation coefficients are outside the confidence interval. This behaviour evokes a pure stationary AR (2) process.

The postulated univariate ARIMA (2,0,0) model was estimated using the maximum likelihood method in EViews software. Various lagged variables of new COVID cases were then entered into the base model in a stepwise regression modelling framework. New COVID cases were aggregated on the weekly, biweekly, and monthly time frames and with different time lags. Finally, the aggregated biweekly new COVID cases with one-week time lag were the only significant predictor of weekly new COVID deaths. This means that the number of weekly new coronavirus deaths is a function of the number of new confirmed cases in the second and third weeks before. The same procedure was adopted to identify the lagged effects of weekly intercity traffic on the new weekly confirmed COVID deaths. The only significant regressor was the weekly traffic flow with a time lag of five weeks. The developed multivariate COVID mortality forecasting model is detailed in Table 3.

**Table 3:** Weekly new COVID deaths forecasting models.

			ARIMA		ARIMAX	
			Coef.	t-stat	Coef.	t-stat
*Explanatory variables*						
Biweekly new confirmed cases (*β*_1_) (*10^−4^) (Time lag: 1 week)			-	-	70.018	5.12
Weekly intercity traffic (*10^−6^) (*β*_2_) (Time lag: 5 weeks)			-	-	36.881	2.05
AR (1)			1.694	26.94	1.663	25.92
AR (2)			-0.795	-13.29	-0.745	-12.23
Constant			1154.355	5.29	-	-
*Descriptive statistics*						
Length of series			70		70	
Log-likelihood			-456.01		-431.07	
*Accuracy (within-sample)*						
Bayesian information criterion (BIC)			13.27		12.99	
Mean absolute % error (MAPE)			**21.37**		**9.49**	
Mean absolute deviation (MAD)					99.29	
Root mean square error (RMSE)			39.20		129.39	
**R** ^**2**^			**0.961**		**0.971**	
*Diagnosis Check of Residuals*						
Ljung and Box (LB) (K = 18)			6.45	-0.59	8.56	-0.45
Kolmogorov-Smirnov (KS)			0.11	2.22	0.07	<1.46
*Forecast Accuracy*						
Out of sample MAPE (%)			**51.46**		**9.183**	
*Likelihood ratio test* (H_0_: *β*_1_, *β*_2_ = 0)			49.88	>	*χ*^2^_*α* = .01,*df* = 2_ = 10.60	

Results illustrate that all the explanatory variables in both models are significant predictors of the dependent variable at a 95% confidence level. Besides, the exogenous variables represented a low VIF equal to 1.14 in the ARIMAX model, indicating no collinearity problem. Moreover, the coefficient of determination of the univariate ARIMA (2,0,0) model is 0.96, which denotes that the developed model adequately reconstructs the observations in the training sample. However, the forecast accuracy of the univariate model is not acceptable regarding the high value of the MAPE criterion in the test sample. In contrast, the ARIMAX model’s out-of-range prediction power is proper as its MAPE criterion is around 9% in the test sample. The ARIMAX model outperforms its univariate counterpart. Since the MAPE criterion of the ARIMA model is halved in the training sample and reduced by 82% in the test sample when the lagged effects of new confirmed COVID cases and intercity traffic were considered exogenous variables. Moreover, the likelihood ratio test also confirms the significance of the partial contribution of the two exogenous variables in the ARIMAX model. The intercity traffic represents a coefficient value of 36.9 which states that each one million higher intercity traffic (≅**9**% * **average weekly traffic**) is associated with 37 more weekly new COVID deaths (≅**3**% * **average weekly new deaths**) with a time lag of 5 weeks. The coefficient of biweekly new confirmed cases also suggests that each ten thousand biweekly new COVID cases (≅**8**% * **average biweekly new cases**), on average, is associated with 70 weekly new COVID deaths (≅**5**% * **average weekly new deaths**) with a time lag of one week, if other factors remain constant.

**Fig 5:**
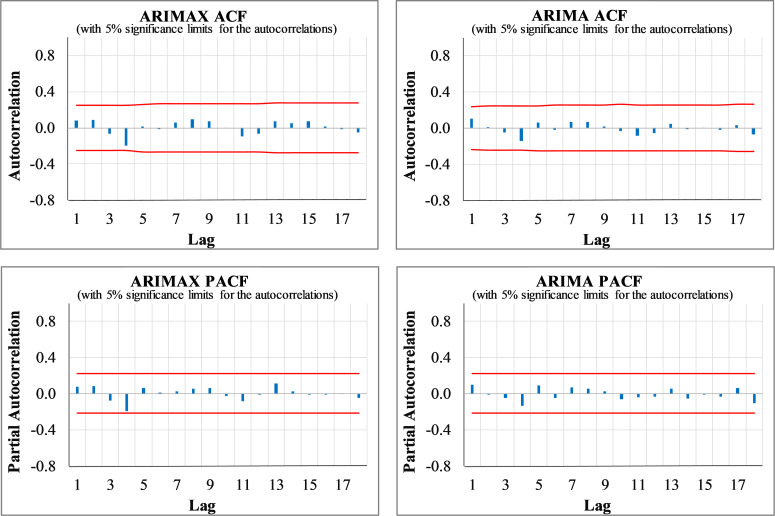
The residuals ACF/PACF plots of the ARIMAX and ARIMA weekly COVID deaths prediction models.

The diagnostic check to validate the adequacy of the developed models showed that they are adequate for the time series forecasting of weekly new COVID deaths in Iran, as is evident in the plots of residuals’ ACF/PACF shown in Fig 5. The figure indicates no significant ACF/PACF coefficients, which means no serial correlation exists among the residuals. Besides, the LB test validates the overall adequacy of the models since the test’s null hypothesis is not rejected, as tabulated in Table 3. The KS test rejects the normality assumption of the distribution of the error term in the univariate model at a 5% significance level. On the contrary, the hypothesis of Gaussian white noise distribution for the ARIMAX model’s residuals is confirmed as the null hypothesis of the test is not rejected at a 99% confidence level. This suggests that taking the two regressors of new COVID cases and intercity traffic into account is vital in understanding the COVID deaths trends.

### Dynamic regressions for weekly COVID new cases time-series

**Fig 6:**
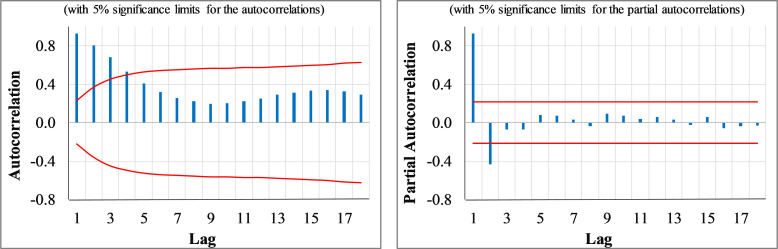
The ACF and PACF plots of the original weekly new confirmed COVID cases time series.

Fig 1 shows the weekly time trends of new COVID confirmed cases which follow nearly the patterns in the intercity traffic time series. The off-peaks of the intercity traffic almost triggers off-peaks of COVID cases with some time lag. Moreover, no significant non-stationarity in the mean or variance is evident. The ADF test was also employed to investigate stationarity. The hypothesis test did not reject the null hypothesis of non-stationarity at a 95% confidence level (see Table 1). Besides, the ACF plot of the original series confirms the stationarity as only the first lags coefficients are significant (see Fig 6).

Regarding the model identification, the ACF plot of the weekly COVID new cases time series in Fig 6 illustrates an exponential decay. Besides, the PACF plot tails off after two significant spikes at the first lags, suggesting that the series follows a pure AR (2) process. To ensure the adequacy of the identified model, various competing ARIMA models were developed and compared based on the parsimony, the significance of the parameters, and the lowest BIC values (See S1 Table). Finally, the best fit model with significant parameters was the ARIMA (2,0,0) model and its ARIMAX extension. The developed models are detailed in Table 4.

**Table 4:** Weekly new COVID cases forecasting models.

			ARIMA		ARIMAX	
			Coef.	t-stat	Coef.	t-stat
*Explanatory variables*						
Weekly intercity traffic (*10^−6^) (*β*_1_) (Time lag: 2 weeks)			-	-	1273.35	1.73
AR (1)			1.599	30.45	1.607	28.07
AR (2)			-0.675	-11.12	-0.683	-10.49
Constant			44078.87	2.47	30274.39	1.65
*Descriptive statistics*						
Length of series			70		70	
Log-likelihood			-732.32		-731.39	
*Accuracy (within-sample)*						
Bayesian information criterion (BIC)			21.167		21.100	
Mean absolute % error (MAPE)			**14.195**		**14.061**	
Mean absolute deviation (MAD)			5076.135		5075.110	
Root mean square error (RMSE)			8240.641		8185.221	
**R** ^**2**^			**0.952**		**0.954**	
*Diagnosis Check of Residuals*						
Ljung and Box (LB) (K = 18)			16.18	0.01	16.09	0.18
Kolmogorov-Smirnov (KS)			0.13	1.65	0.11	1.71
*Forecast Accuracy*						
Out of sample MAPE (%)			**64.12**		**63.93**	
*Likelihood ratio test*			1.86	<	*χ*^2^_*α* = .05,*df* = 1_ = 5.024	

Reviewing Table 4, all the independent variables are statistically significant at a 10% significance level. Moreover, the ARIMA model indicates an R-squared value of 0.952, which denotes that over 95% of the variations in the current weekly new COVID cases are explained only by considering the serial correlations in the underlying time series. Besides, the multivariate ARIMAX model outperforms the univariate model slightly, regarding the increase in the coefficient of determination and the log-likelihood value. Nonetheless, the likelihood ratio rejects the hypothesis of statistical significance of the fit superiority of the ARIMAX model at a 5% significance level. Meanwhile, the significant coefficient value of the weekly intercity traffic demonstrates that each one million more intercity traffic is associated with an approximate 1273 more weekly new confirmed COVID cases after two weeks, given that other influential factors remain constant. Moreover, the MAPE criterion demonstrates that the developed instruments properly reconstruct observations with only about 14% mean absolute error. On the other hand, the out-of-range prediction power of the Box-Jenkins methodology was not appropriate based on the MAPE criterion, although the developed model performs well in the short-term predictions. This is not surprising as the influential factors on the COVID trends are not limited to the intercity traffic pattern. Considering factors such as the percentage of compliance with health protocols will strengthen the model’s ability to make medium-term predictions based on causal relationships if such data is available.

**Fig 7:**
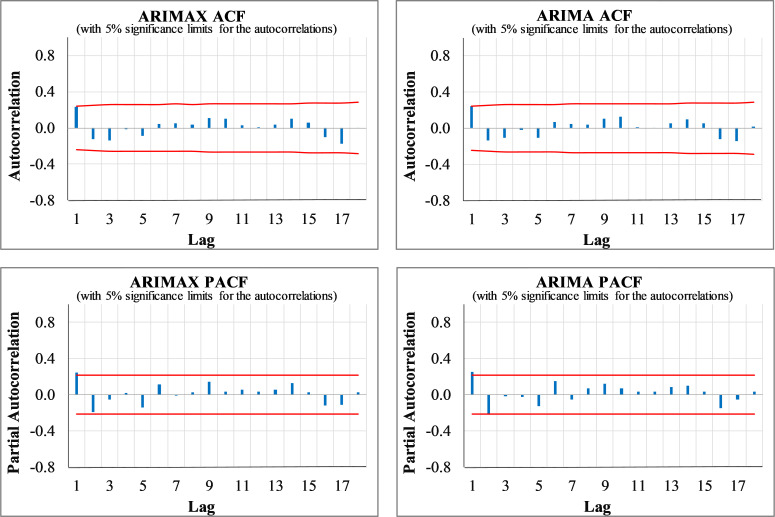
The residuals ACF/PACF of ARIMA and ARIMAX models (new weekly COVID cases).

Finally, the model diagnosis checking is conducted by testing the white noise characteristics of the residuals in terms of non-autocorrelation, zero mean, and stationarity in the variance. The ACF/PACF plots of the residuals do not represent any significant coefficients at a 5% significant level except the first lag (see Fig 7). Besides, the LB and KS test results also confirm the white noise characteristics of the residuals for both models (see Table 4).

## Discussion

Drawing on the COVID trends and loop detectors’ intercity traffic datasets, this research contributes the first empirical study on the dynamic association of intercity travel patterns and COVID spread in Iran. This research also evaluates a new non-pharmaceutical intervention (NPI) with economy-friendly features applicable for Low-to-Middle-Income Countries (LMICs). The most important contribution of this paper is its concentration on the associations between several lagged effects of intercity traffic and COVID trends. Realizing these delayed effects provides crucial information for health authorities to evaluate the effectiveness of the human mobility restriction policies on containing the pandemic. The study also compared the ARIMAX modelling framework with its univariate counterpart based on the in-sample and out-of-range forecast accuracy measures.

The results indicated that a one million increase in the weekly intercity traffic triggers a 2% (1273 new weekly COVID cases) increase in the weekly new confirmed COVID cases with a time lag of two weeks which confirms the previous findings. Wang et al. [39] studied the relationships between COVID spread and human mobility in Australia. They reported a 7 to 14 days period between lockdown policy implementation and the reduction in the virus spread. Zhu et al. [40] investigated the mediation effects of air quality on the association of human mobility and COVID infections in China. The results indicated that a unit increase in the human mobility index is associated with a 6.45% increase in daily COVID confirmed cases at lag 0–14. Another study also reported that the effects of social distancing on decreasing transmission are not perceptible for at least 9–12 days after implementation [38]. The authors stated that this lag time would reflect the time for symptoms to manifest after infection, worsen, and be reported [38]. Moreover, this two-week lag time might also be related to the incubation period of the disease, which has been reported in various epidemiological studies to have a maximum of 14 days [50, 51].

The results also illustrated that each one million intercity traffic is associated with a 3% increase (37 weekly new infections) in the weekly new confirmed COVID deaths with a time lag of 5 weeks. These results are in line with the literature findings, which almost reported a delay of around 21 days between new COVID infections and deaths [52, 53]. Moreover, based on the findings of the current study, a time lag of 2 weeks was detected between an increase in the intercity traffic and the rise in new confirmed COVID cases. Hence, a time lag of 5 weeks between the intercity traffic and COVID deaths is interpretable and reasonable. Moreover, each ten thousand biweekly new confirmed COVID cases were associated with 70 weekly new COVID deaths (≅**5**% * **average weekly new deaths**) with a time lag of one week. It should be noted that the biweekly new COVID cases were associated with new weekly COVID deaths, meaning that an average of two-week periods exists between the new COVID infections and deaths. Based on the literature, it was expected that a longer lag time would have been seen. Nonetheless, recent studies indicated that in countries that have not incorporated adequate measures to contain the outcomes of the disease, shorter periods, even negative time lags between the infections and deaths’ peaks, are pretty reasonable [54].

The interrupted time series analysis framework was employed to investigate the direct contribution of the STB policy in the reduction of intercity travels and also COVID infections/deaths trends. The intervention variable didn’t indicate a significant association between the STB policy implementation and COVID new cases and deaths time trends. On the other hand, the interrupted time series analysis revealed that the policy declines the intercity traffic by 29%. Besides, the significance of the lagged effects of weekly intercity traffic on COVID trends was confirmed in this research. Although the current fines have reduced intercity travel during the intensification periods of the pandemic, this reduction is not sufficient. So, the results strongly recommend that health authorities increase the enforcements, considering the significant associations between the intercity travels and COVID spread. Moreover, adherence to health protocols and social distancing should be adopted in conjunction with the STB policy to achieve acceptable results in controlling the epidemic. In line with this statement, a recent study reported that the state-level mobility restrictions in the US, which dropped the mobility level by 35–63% relative to the usual conditions, were less effective in containing the COVID transmission compared with the individual-level social distancing behaviours [38]. Nonetheless, if such policies were not adopted, the disease transmission would have been evolved on a much more worrying trend.

This research also introduced dynamic multivariate COVID forecasting tools for Iran’s epidemic time trends. The weekly new COVID mortality time series indicated that the ARIMAX model outperforms the univariate ARIMA model based on the MAPE criterion. The MAPE criterion of the univariate model was halved in the training sample and reduced by 82% in the test sample when the lagged effects of intercity traffic and new confirmed COVID cases were considered as exogenous variables. Consequently, the ignorance of existing causal links with explanatory variables in the univariate models would result in biased estimates, especially in the out-of-sample predictions. On the other hand, in the case of weekly new COVID cases time series, the ARIMAX model with the intercity traffic as the only exogenous regressor did not perform better compared with the univariate model. Accounting for the variables such as the percentage of compliance with health protocols might improve the predictive power of the models; If such information is available.

There are some limitations in this research, which should be considered when interpreting the results. First, the information on human mobility was not available for different travel modes and purposes. Analyzing the contribution of each intercity travel mode in the transmission of the disease would bring valuable insights for policymakers. Second, no data was available on the percentage of community compliance with health protocols. Undoubtedly, if such a crucial factor is considered, in addition to increasing the accuracy of the models, it will be possible to evaluate the efficiency of the STB policy in different levels of compliance with health protocols. Third, although the statistical models indicated appropriate forecasting performance, advanced machine learning methods might better capture the nonlinear effects in the COVID time series.

## Conclusion

Drawing on the weekly new confirmed COVID cases/deaths and intercity highways traffic datasets, this study presents national-level empirical research to examine how the changes in intercity travel patterns are adherent to the STB policy and how such changes affect COVID spread. The following were arrived at in this research: (1) the STB policy declined the intercity travel significantly by around 29%. Moreover, the findings confirm a significant association between the traffic pattern and COVID trends. Nevertheless, this reduction in human mobility is not well enough to have an evident effect on COVID mortalities or confirmed COVID infections. (2) A one million increase in the weekly intercity traffic triggers a 2% increase in the weekly new confirmed COVID cases with a time lag of two weeks which is interpretable regarding the 14 days incubation period. (3) Each one million intercity traffic is associated with a 3% increase in the weekly new confirmed COVID deaths with a time lag of 5 weeks. The 3-weeks lag time between COVID infections and deaths in the literature and the 2-weeks incubation period confirm this finding. (4) The multivariate ARIMAX method outperforms the univariate Box-Jenkins approach in the COVID mortality prediction model, accounting for the simultaneous impacts of lagged variables of intercity traffic and new confirmed COVID cases. Indeed, neglecting these causal associations in the model development results in the model misspecification, which in turn triggers biased estimates of future COVID time trends, especially in out-of-sample forecasts.

The developed models could be utilized by transport authorities and health policymakers to predict future time trends of the COVID pandemic and evaluate the effectiveness of newly implemented interventions. Besides, the study introduces the ARIMAX method as a valuable framework to investigate the explicit effects of a specific contributing factor on COVID trends; while controlling for underlying autocorrelation and dynamic impacts of other exogenous variables.

Although the Box-Jenkins method efficiently captured the linear dependencies in the COVID time series, the COVID observations would also be spatially correlated. The results highlighted the importance of intercity human mobility variations on COVID disease spread. These findings suggest that the spatial dependencies might cause COVID infections in spatial proximity to have similar spread intensity. Nonetheless, the current literature on applying statistical and machine learning techniques to COVID data considers the observations as independent components, which might result in biased parameter estimates and inferences. Future research should shed light on these spatial associations, adopting advanced deep neural network models, such as Graph Convolutional Networks (GCN). Moreover, a richer problem space with more exogenous variables at the disaggregate level would bring further insights on potential differences between provinces. As the health infrastructures differ between provinces, the province-level economic attributes might have a moderation effect into the association of human mobility with COVID deaths trends.

## Supporting information

S1 Table Weekly new COVID cases forecasting models, comparing ARIMA alternatives. (DOCX)

S1 File Weekly time trend of intercity traffic flow, and COVID trends. (XLSX)
