# Supplementary material for: How do the smart travel ban policy and intercity travel pattern affect COVID-19 trends? Lessons learned from Iran
Source: PLoS One. 2022 Oct 18;17(10):e0276276. doi: 10.1371/journal.pone.0276276 (PMC9578609; doi:10.1371/journal.pone.0276276)
Supplement: S1 Table — (DOCX) [file pone.0276276.s001.docx]

**S1 Table. weekly new COVID cases forecasting models, comparing ARIMA alternatives.**

|  |  |  | ARIMA (2,0,0) | | | | ARIMA (1,0,2) | | ARIMA (3,0,0) | | ARIMA (0,0,3) | |
| --- | --- | --- | --- | --- | --- | --- | --- | --- | --- | --- | --- | --- |
|  |  |  | Coef. | | t-stat | | Coef. | t-stat | Coef. | t-stat | Coef. | t-stat |
| *Explanatory variables* | | | |  | |  |  |  |  |  |  |  |
| AR (1) | | | | 1.599 | | 30.45 | 0.872 | 11.45 | 1.846 | 22.21 |  |  |
| AR (2) | | | | -0.675 | | -11.12 |  |  | -1.252 | -7.77 |  |  |
| AR (3) | | | |  | |  |  |  | 0.357 | 1.63 |  |  |
| MA (1) | | | |  | |  | 1.025 | 7.17 |  |  | 1.914 | 18.44 |
| MA (2) | | | |  | |  | 0.503 | 2.45 |  |  | 1.745 | 13.50 |
| MA (3) | | | |  | |  |  |  |  |  | 0.682 | 7.84 |
| Constant | | | | 44078.87 | | 2.47 | 42615.71 | 2.08 | 42486.49 | 1.97 | 44139.22 | 6.26 |
|  | | | |  | |  |  |  |  |  |  |  |
| *Descriptive statistics* | | | |  | |  |  |  |  |  |  |  |
| Length of series | | | | 70 | |  | 70 |  | 70 |  | 70 |  |
| Log-likelihood | | | | -732.32 | |  | -736.94 |  | -740.51 |  | -744.53 |  |
|  | | | |  | |  |  |  |  |  |  |  |
| *Accuracy (within-sample)* | | | |  | |  |  |  |  |  |  |  |
| Bayesian information criterion (BIC) | | | | 21.167 | |  | 21.273 |  | 21.386 |  | 21.576 |  |
| Mean absolute % error (MAPE) | | | | **14.195** | |  | **16.035** |  | **15.426** |  | **22.56** |  |
| Mean absolute deviation (MAD) | | | | 5076.135 | |  | 4896.017 |  | 5164.120 |  | 7359.18 |  |
| Root mean square error (RMSE) | | | | 8240.641 | |  | 8782.106 |  | 8958.366 |  | 10087.17 |  |
| $\mathbf{R}^{\mathbf{2}}$ | | | | **0.952** | |  | **0.948** |  | **0.956** |  | **0.934** |  |
|  | | | |  | |  |  |  |  |  |  |  |
| *Diagnosis Check of Residuals* | | | |  | |  |  |  |  |  |  |  |
| Ljung and Box (LB) (K=18) | | | | 16.18 | | 0.01 | 5.99 | 0.22 | 6.62 | 0.35 | 62.82 | 0.59 |
| Kolmogorov-Smirnov (KS) | | | | 0.13 | | 1.65 | 0.18 | 2.64 | 0.17 | 1.70 | 0.12 | 2.31 |
|  | | | |  | |  |  |  |  |  |  |  |
| *Forecast Accuracy* | | | |  | |  |  |  |  |  |  |  |
| Out of sample MAPE (%) | | | | **64.12** | |  | **65.35** |  | **70.56** |  | **67.29** |  |
|  | | | |  | |  |  |  |  |  |  |  |
